# A commentary on ‘Metal ureteral stents for ureteral stricture: 2 years of experience with 246 cases’ (Int J Surg 2024;110:66–71)

**DOI:** 10.1097/JS9.0000000000001374

**Published:** 2024-03-25

**Authors:** Binsen Li, Tongxin Yang

**Affiliations:** Department of Urology, The Second Affiliated Hospital of Kunming Medical University, Wuhua District, Kunming, Yunnan, People’s Republic of China

*Dear Editor,*

Ureteral stricture is reckoned as a challenging and complicated urological disease. It can cause multiple complications, such as hydronephrosis, renal dysfunction, etc. With the development of ureteral reconstructive and repair surgery, endoscopy, and ureteral stents, self-expanding stent technology has been proven to provide significant benefits to patients with ureteral strictures.

Complications associated with metallic ureteral stent (MUS) placement can be fatal to the recovery of patients with ureteral stricture or ureterovaginal fistula. Numerous studies have reported stent migration as a common complication. A study by Weinberger *et al*.^1^ reported 10 cases that received MUS placement after kidney transplantation, of which 4 out of 10 (40%) experienced stent migration within 3 months after MUS placement. Another study by Moskovitz *et al*.^2^ reported 49 cases treated with MUS for various conditions, with 7 out of 49 (14%) experiencing stent migration. Moreover, as Professor Xin Wei *et al*. reported, stent migration occurred in 53 of 247 (21%) cases with different conditions. We consider the high incidence of stent migration, which suggests that the ureteral stents may lack an anchoring mechanism. Therefore, we would like to inquire about our experiences in preventing MUS migration. Another aspect is the use of tandem ureteral stenting for ureteral obstruction, which was reported years ago. A study of 104 cases by Liu *et al*.^3^ found that tandem ureteral stents had longer patency compared to single stents (*P*=0.022). As Professor Xin Wei *et al*. mentioned, tandem stents may also be effective against ureteral obstruction. We would like to inquire about the success rate of tandem stenting in managing obstruction in their study. In addition, Professor Xin Wei *et al*. mention that ‘The ureter is usually shorter after kidney transplantation. By using a 10cm long stent to support the entire ureter, the stent migration rate can be reduced.’ We would like to know the mechanism behind stent migration when the stent supports the entire length of the ureter and the value of a shorter stent specially designed for short ureters.

We noticed that all cases managed by Professor Xin Wei *et al*. with migrated metallic ureteral stents (MUS) were successfully treated. In another report, Professor Xin Wei and colleagues described exchanging 9 stents and repositioning 30 migrated stents back into the normal position, with all stents remaining patent until the last follow-up^4^. The success rate for repositioning migrated stents was high in their study. We would inquire about the indications they used to decide between stent repositioning versus full stent replacement in migrated MUS cases. And We wonder how it could reposition benefit patients.

Ureteral balloon dilation is considered to help endoscopic or stenting instruments to pass the stricture. In a report by Kuntz *et al*.^5^, 151 cases had undergone balloon dilation, the success rate was 95% (143/151), and only 8 cases had intra-operation complications (4 mucosal splitting/tear; 3 perforations; 1 lost access), and they reported that balloon dilation was associated with a high success rate. In the report by Professor Xin Wei and colleagues, ureteral balloon dilation was utilized to manage ureteral narrowing. We would inquire as to the number of cases that underwent the balloon dilation and the outcomes of follow-up after utilizing this technique.

Ureterovaginal fistula is a complication of radical pelvic surgery, with an incidence ranging from 0.9 to 2%^6^. The traditional management of ureterovaginal fistulas is ureteroneocystostomy. Puntambekar *et al*. reported five cases of ureteroneocystostomy, two of which were patients with ureterovaginal fistula. The outcomes were excellent, and no postoperative complications were noted. All patients remained symptom-free at 6 months after surgery^7^. Several researchers have reported successful outcomes as well. Therefore, it can be concluded that the techniques of ureteroneocystostomy are mature. MUS placement is another option with good outcomes and mature techniques. As Selzman *et al*.^8^ reported, the ureteral stents were used in eight women with ureterovaginal fistulas for 4–8 weeks (mean 5.5), and seven of them had their fistula cured. Since both ureteroneocystostomy and MUS placement are effective in resolving the fistula, it would be helpful to know which one is better and how to choose between them in different clinical situations. Fistulas resulting from radiation therapy pose an even greater challenge, as this type of fistula and associated ureteral strictures are complex. Therefore, ureteroneocystostomy can be difficult to implement in such cases. In this situation, we wonder about the short-term success rates and long-term outcomes of MUS placement.

In summary, we sincerely appreciate the excellent contributions made by Professor Xin Wei *et al*. in investigating self-expanding metallic stents for ureteral strictures and ureterovaginal fistulas. They took a significant step forward in this important topic. Self-expanding stent technology provides minimally invasive and effective treatment options for these conditions with obvious advantages. However, many specific operative details and long-term outcomes still need to be further elucidated through prospective studies and high-quality case-controlled comparative studies. We look forward to more research results in the future to better guide the precise application of this novel technology in the management of complex post-transplant complications. Again, we thank Professor Xin Wei and colleagues for their constructive research work. We sincerely hope that they will continue contributing and shedding light on the path forward for advancements in this field.

## Ethical approval

Ethical approval is not required.

## Consent

Consent is not required.

## Sources of funding

None.

## Author contribution

B.L.: writing, data collection, and analysis; T.Y.: study design.

## Conflicts of interest disclosure

The authors declare that they have no conflicts of interest.

## Research registration unique identifying number (UIN)

None.

## Guarantor

Tongxin Yang.

## Data availability statement

The data presented in this commentary are all derived from the referenced literature. Specific data and analysis results can be found in the cited references.

## Provenance and peer review

Commentary, internally reviewed.
